# Approaching the potential of model-data comparisons of global land carbon storage

**DOI:** 10.1038/s41598-019-38976-y

**Published:** 2019-03-04

**Authors:** Zhendong Wu, Gustaf Hugelius, Yiqi Luo, Benjamin Smith, Jianyang Xia, Rasmus Fensholt, Veiko Lehsten, Anders Ahlström

**Affiliations:** 1 Department of Physical Geography and Ecosystem Science, Lund University, Sölvegatan 12, SE-223 62 Lund, Sweden; 2 Department of Geosciences and Natural Resource Management, University of Copenhagen, 1350 Copenhagen, Denmark; 3 Department of Earth System Science, School of Earth, Energy and Environmental Sciences, Stanford University, Stanford, CA 94305 USA; 4 Department of Physical Geography and Bolin Centre for Climate Research, 10691 Stockholm University, Stockholm, Sweden; 5 Center for Ecosystem Science and Society (Ecoss) and Department of Biological Sciences, Northern Arizona University, Flagstaff, Arizona USA; 6 Hawkesbury Institute for the Environment, Western Sydney University, Locked Bag 1797, Penrith, NSW 2751 Australia; 7 Research Center for Global Change and Ecological Forecasting, School of Ecological and Environmental Sciences, East China Normal University, Shanghai, China; 8 Institude of Eco-Chongming (IEC), 3663 N. Zhongshan Rd., Shanghai, 200062 China; 9 Swiss Federal Institute for Forest, Snow and Landscape research (WSL), Zürcherstr, 11 CH-8903 Birmensdorf, Switzerland; 10 Center for Middle Eastern Studies, Lund University, Box 201, SE-221 00 Lund, Sweden

## Abstract

Carbon storage dynamics in vegetation and soil are determined by the balance of carbon influx and turnover. Estimates of these opposing fluxes differ markedly among different empirical datasets and models leading to uncertainty and divergent trends. To trace the origin of such discrepancies through time and across major biomes and climatic regions, we used a model-data fusion framework. The framework emulates carbon cycling and its component processes in a global dynamic ecosystem model, LPJ-GUESS, and preserves the model-simulated pools and fluxes in space and time. Thus, it allows us to replace simulated carbon influx and turnover with estimates derived from empirical data, bringing together the strength of the model in representing processes, with the richness of observational data informing the estimations. The resulting vegetation and soil carbon storage and global land carbon fluxes were compared to independent empirical datasets. Results show model-data agreement comparable to, or even better than, the agreement between independent empirical datasets. This suggests that only marginal improvement in land carbon cycle simulations can be gained from comparisons of models with current-generation datasets on vegetation and soil carbon. Consequently, we recommend that model skill should be assessed relative to reference data uncertainty in future model evaluation studies.

## Introduction

Terrestrial ecosystems can be a sink or a source of carbon (C) depending on the balance between primary production (C-influx) and the rate of return of vegetation and soil C to the atmosphere through respiration, biomass burning, and other minor release fluxes (C turnover). The C turnover may be characterized by average residence time (the total C stock divided by the total output flux) before being released back to the atmosphere^1^. The Fifth Assessment Report of the Intergovernmental Panel on Climate Change (IPCC AR5) reported that a majority of Earth System Models (ESMs) project a continued net C uptake under all future CO_2_ emission scenarios, yet some models simulate a net C emission due to the combined effect of climate change and land use change^2^. Different process representations both of C-influx and residence time in different models have been demonstrated to explain these model differences^3^.

Previous studies found that the large spread (e.g., vegetation carbon increased by from 52 to 477 Pg C with 4 °C of global warming^3^) of the predicted future vegetation biomass among models was mainly a result of inter-model differences in the residence time of vegetation C^3,4^. Todd-Brown, *et al*.^5^ pointed out that the large disagreement (ranged from 510 to 3040 Pg C) on the global distribution of soil C across models was primarily due to differences in the representation of Net Primary Production (NPP) and turnover time of soil C. The relative contributions of different carbon cycle processes to such model-data disagreement is currently not known. Three major factors may contribute to the model-data disagreement, (i) poor representation of ecological processes in the model, e.g., C assimilation, vegetation and soil turnover, (ii) uncertainties in, and the resolution of, data used to force models. Such data are not limited to climatic data but also include environmental data (e.g., information on land use), and (iii) uncertainties in present reference data from empirical observations, e.g. maps of aboveground biomass (AGB, the C stored in leaf and woody compartments) and soil C storage, against which model improvements are evaluated. If the uncertainties in these empirical datasets are large it may be difficult to assess the true state and thus difficult to detect model improvements when comparing model outputs to empirical datasets. Therefore, a model improvement should be assessed relative to the reference data uncertainty.

Vegetation and soil C stocks are dependent on the influx of C from Gross Primary Production (GPP), for which estimates differ between models^6^ and between models and empirical datasets^7,8^. C turnover from vegetation and soil is likewise simulated differently by present models, both in terms of the represented processes and their scaling (i.e. the turnover rate, which may be fixed or variable depending on forcing and system state)^3–5^. This study discriminates the role of C-influx and C turnover in terms of their effect on the agreement between model-simulated carbon exchange and storage, and empirical data products. To this end we used the Traceability Framework (TF^1,9,10^) to represent the structure and carbon dynamics of a global dynamic ecosystem model, LPJ-GUESS^11,12^. The model is one of the so-called second generation of such models^13^ that incorporates detailed representation of plant individual, demographic and landscape processes governing vegetation carbon turnover, as well as soil carbon and nutrient cycles^12^. It has been extensively applied and evaluated in multiple studies^14,15^.

The TF method preserves the model structure and carbon dynamics in space and time and allows us to replace the model-simulated fluxes, i.e. C-influx, vegetation C turnover rate, and soil C turnover rate, with estimates from empirical datasets and products derived by combining empirical datasets. By comparing outputs before and after replacing a simulated flux with an empirically-estimated one, we can identify the processes (e.g., photosynthesis or vegetation turnover) responsible for discrepancies between model predictions and observation-based estimates.

## Methods

### Traceability Framework

**Figure 1:**
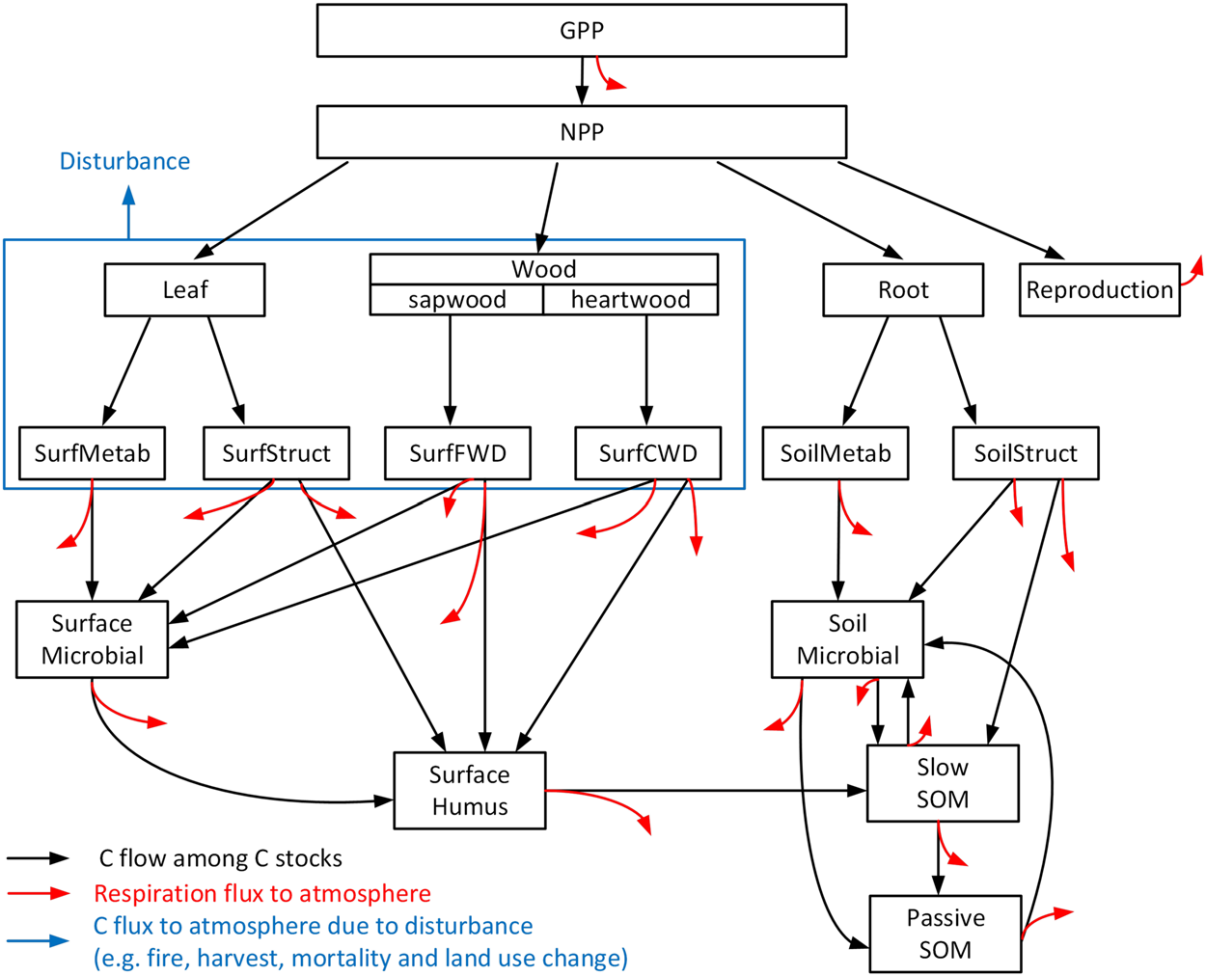
Schematic representation of the C transfers among multiple stocks in vegetation and soil (including litter) in LPJ-GUESS. The traceability framework adopts the same structure and preserves all relative flows between C stocks.

The TF decomposes the full structural complexity of a biogeochemical system model into traceable components (e.g., NPP, allocation coefficients, transfer coefficients etc.), based on mutually independent properties of modeled biogeochemical processes, so that modeled responses of the terrestrial C cycle to climate change and disturbances can be better understood^1,9,10^. Most process-based models, such as LPJ-GUESS^11,12^, represent the carbon cycle as a network of stocks, fluxes, and transfer rates (Fig. 1). These components (e.g., C stocks, C-influx and transfer coefficients among stocks) can be reconstructed mathematically as a linear system using the TF^1,9,10^. The mathematical framework (see details in Supplementary Text 1) converts the transfer of C between individual stocks into a set of equations, preserving relative flows and turnover rates for each location (globally) and year^16^. Here the TF is constructed from results of a fully dynamic simulation by LPJ-GUESS over the years 1901–2014. This historical simulation uses climate information from the CRUNCEP version 7^17^, historical CO_2_^18^, nitrogen deposition^19^ and transient land use data^20^ as forcing (details of the simulation protocol can be found in Supplementary Text 2).

The LPJ-GUESS TF fully represented the dynamics for all C stocks as simulated by LPJ-GUESS. However, because of the individual-based representation of vegetation (with stochastic mortality) and the nitrogen dynamics of LPJ-GUESS, some stocks in the model do not reach a fully reproducible steady state after initialization. As a result, the TF implementation shows a small offset in the passive soil carbon stock after initialization in 1901. This offset is comparably small and does not significantly influence the results presented here (Supplementary Fig. S1).

### Implementation of model-data fusion

The TF allows us to isolate the impact of C-influx and turnover on C stocks and flux dynamics by replacing model-simulated C-influx and C turnover rates with empirical datasets and products derived by combining empirical datasets. Here we refer to this method as model-data fusion, and the individual data-model combinations are referred to as TF-realizations. The model-data fusion was implemented in three different ways: **(i)** by only replacing the C-influx component with observation-based GPP and NPP products; **(ii)** by only replacing the C turnover rate (includes vegetation turnover rate alone, soil turnover rate alone, and both); **(iii)** by replacing the C-influx and C turnover rates together. The details of the implementation of the model-data fusion are given in Supplementary Text 3.

Five observation-based C-influx datasets were fused into the TF representation of LPJ-GUESS. These datasets are used extensively for model evaluation and carbon cycle analysis, and are considered the state-of-the-art in current quantification of biospheric productivity across the global land surface^6,8^. The datasets employed where the remotely-sensed NPP product from MODIS (Moderate-resolution Imaging Spectroradiometer) MOD17 v55 (2000–2014)^21^ and four global flux tower-based GPP products from FLUXCOM (1982–2011)^22,23^. MODIS NPP was calculated from removing maintenance and growth respiration from GPP estimated by Light Use Efficiency (LUE) model translating absorbed Photosynthetically Active Radiation (APAR) to actual productivity. The four GPP products were derived from eddy covariance measurements and subsequently upscaled to global scale by the use of Model Trees Ensembles (MTE^24^), Artificial Neural Networks (ANN^25^), Multivariate Adaptive Regression Splines (MARS^26^) and Random Forests (RF^27^). FLUXCOM GPP was translated to NPP by removing the plant respiration following the plant respiration to GPP ratio in the fully dynamic LPJ-GUESS simulation. Turnover rates of vegetation and soil C were estimated by combining empirical datasets of vegetation C, soil C, and C-influx. Using C-influx instead of C-efflux will introduce a bias in non-steady state systems, where C-influx does not equal C-efflux, and consequently we refer to the turnover rate as apparent turnover rate. However, since we calculate and correct simulated turnover rates in the same way, we offset at least part of this bias (assuming the model simulates correct trends).

To investigate the role of C-influx on model-data disagreement, the five NPP datasets (derived from MODIS NPP and four FLUXCOM GPP products) were tested for their effect on the downstream C exchange and C stock. We merged simulated and empirical NPP in the year 1982 (FLUXCOM) or 2000 (MODIS) to allow initialization of the C stocks in the TF-realization. The merging and adjustment of the NPP time series preserves the inter-annual variability of the empirical GPP or NPP dataset, but changes its trend to correspond with the simulated trend during the common period (1982–2011 for FLUXCOM and 2000–2014 for MODIS) (Supplementary Text 3). The resulting NPP time series are hereinafter referred to as refined NPP and covers the years 1901–2014. The AGB and soil C estimates resulting from the TF-realizations (where NPP was replaced with refined NPP) were evaluated against the empirical datasets of AGB and soil C.

We further replaced simulated turnover rates of vegetation and soil C. Two empirical datasets of AGB and two datasets of soil C were used in combination with the refined NPP datasets (described above) to create ten datasets each of apparent vegetation and soil C turnover rates. Turnover rates were produced by dividing the NPP with soil C or AGB.

Two independent contemporary estimates of AGB include a satellite Vegetation Optical Depth based estimate of AGB from Liu, *et al*.^28^ based on an empirical relationship between VOD and AGB, hereinafter referred to as VOD-AGB. The other AGB product was originated from the two pan-tropical datasets^29,30^, subsequently integrated in Avitabile, *et al*.^31^, hereinafter referred to as PAN-AGB.

Two datasets of soil C were used in the analysis, the SoilGrids^32^ and the WISE dataset^33^. The SoilGrids data was constructed from machine learning methods by training ~150,000 soil profiles from the ISRIC-WoSIS database, with accounting for remote sensing-based soil covariates, e.g., land cover classes, lithology maps, and climatic images^32^. The WISE data was derived from a statistical analysis of ~21,000 soil profiles from the ISRIC-WISE database, with accounting for possible effects of regional variation in climate on soil properties^33^. We adjusted the two soil C datasets in order to capture the amount of soil C which interacts with vegetation and the C dynamics that is captured by the dynamic model over the simulation period. We adjusted the soil C datasets by removing soil C that is either formed by processes not included in the dynamic model or C that is likely stored under the past climatic conditions that are not represented by the early 20^th^-century climate, and thereby by design not captured in the model initialization. We, therefore, modified the soil C datasets over peatland and permafrost, based on the assumption that vegetation in peatlands only interacts with the upper 40 cm soil, and vegetation in permafrost only interacts with soil within the active layer. The soil C datasets over the other land cover classes were not adjusted. The global active layer thickness was derived from the CLM model^34^, and the peatland fraction derived from Hugelius, *et al*.^35^. This way the soil C datasets, and the apparent turnover rates derived from these datasets, better represent the simulated apparent turnover rates.

Apart from characterizing the role of C-influx and C turnover rate separately, we also explored the interaction effect of C-influx and C turnover rate on the downstream C exchange. We fused the five refined NPP datasets together with the 20 apparent turnover rate datasets into the LPJ-GUESS model, which resulted in 40 TF-realizations in total for all combinations. Thus we ended up with 10 TF-realizations for replacing NPP and vegetation turnover, 10 TF-realizations for replacing NPP and soil turnover, and 20 TF-realizations for replacing NPP and both turnover. In each TF-realization, only one NPP dataset was used for correcting both simulated NPP and turnover rate. Since the empirical datasets on C stocks were used in the calculation of apparent turnover rate, we aggregated the carbon dynamics of the TF-realizations which were corrected for turnover rate globally and annually and compared them to the net land flux from the 2016 Global Carbon Budget, GCB, synthesized by the Global Carbon Project^36^.

### Evaluation

We diagnosed the performance of the TF-realizations using: (1) two independent datasets of AGB, VOD-AGB and PAN-AGB; (2) two independent datasets of soil C to two-meter depth, WISE and SoilGrids; and (3) a global net land flux dataset from GCB^36^, which was calculated from a bookkeeping model. GCB net land flux represents the residual of fossil fuel emissions, atmospheric growth and oceanic uptake of carbon. The details of the reference data are provided in Supplementary Table S1. Three statistical evaluation metrics were used to assess the consistency between the TF-realizations and reference data: Willmott’s Index of Agreement (IoA^37^), Pearson correlation coefficient (r) and Root Mean Square Error (RMSE). IoA was used here to consider general agreement by accounting for both the strength of relationship and similarity in magnitude, where one indicates a perfect agreement and zero indicates complete disagreement. Model-data agreement was evaluated across global land cover classes following Ahlström, *et al*.^38^, Supplementary Fig. S2.

### Limitations to benchmarking

Confidence in model-data evaluations depends on the quality and the confidence that can be assigned to the reference data. We described the problem of limitations to benchmarking conceptually and developed a simple metric of the confidence and quality of the reference data that is based on the agreement between independent contemporary reference datasets that describe the same variable (Supplementary Fig. S3). Conceptually, in this framework, when evaluating model outputs against reference data, there are three possible situations of the outcome of evaluations. (1) model-data agreement is lower than the agreement between the reference data (here referred to as baseline knowledge), which implies that there is a potential to improve models to reach the current baseline knowledge. (2) the model-data agreement is comparable to the baseline knowledge, which means that the model captures the knowledge based on independent data. (3) The model-data agreement is higher than the baseline knowledge. Such agreement may however not necessarily represent a model improvement due to limited understanding of the actual conditions, such as the actual distribution of biomass. In the analysis below we illustrated this by also showing the agreement between the empirical datasets as an estimate of the baseline knowledge, while acknowledging that the metric we used here is simple and may have its limitations.

## Results

### Aboveground biomass

**Figure 2:**
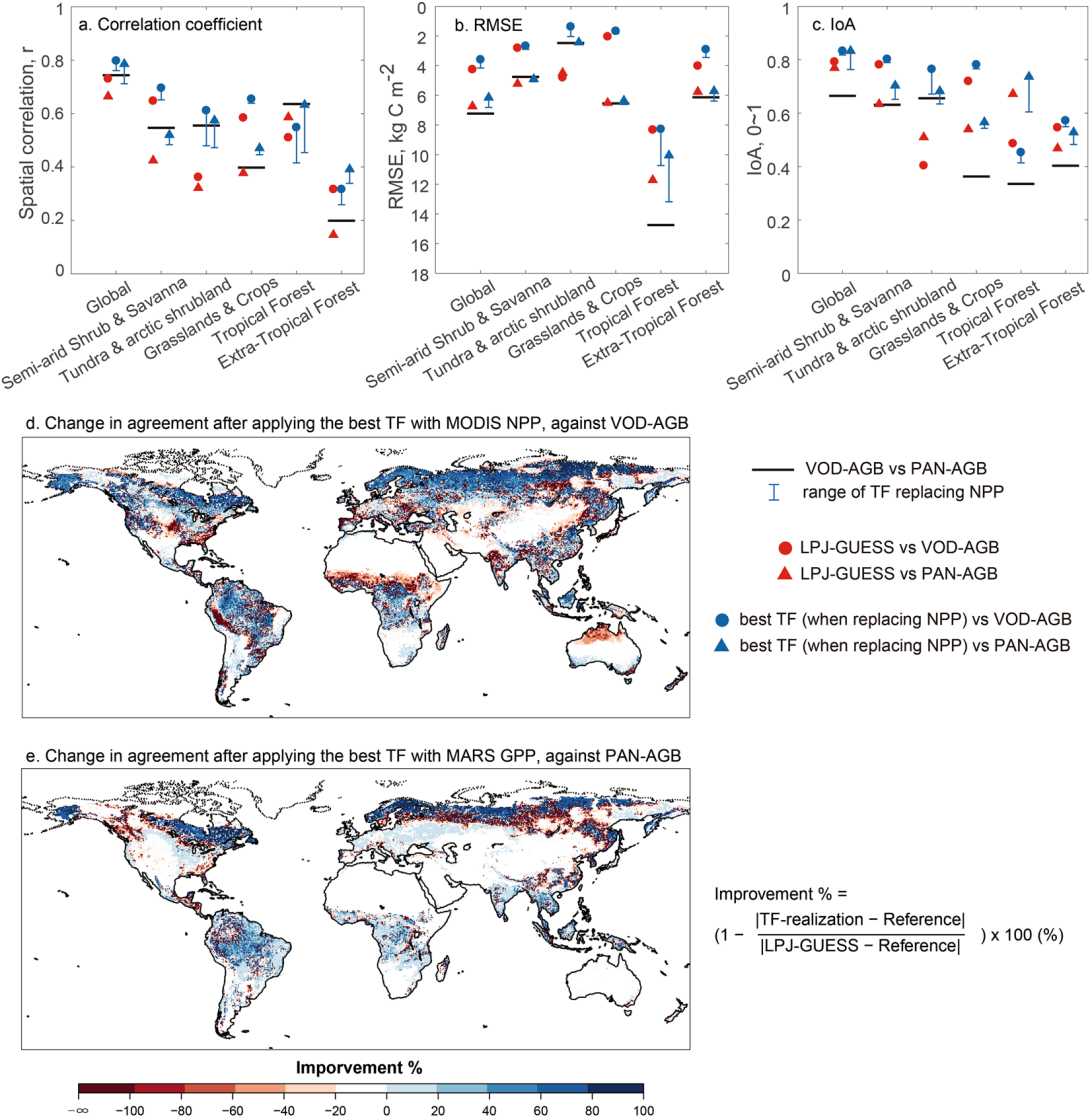
Comparison of predicted AGB with two empirical datasets (panel a–c) over six land cover classes (Supplementary Fig. S2). Black line segments show the comparison between two independent contemporary empirical datasets of AGB. Output from LPJ-GUESS simulation before replacing NPP is marked as red. TF-realizations by replacing simulated NPP with refined NPP derived from MODIS NPP and FLUXCOM GPP are marked as blue. Error bars show the range of results from replacing simulated NPP with the five refined NPP. Panel d and e show the improvement of the best TF-realizations in terms of the magnitude of AGB difference compared to VOD-AGB and PAN-AGB, respectively.

We compared AGB from the TF-realizations where simulated NPP had been replaced by refined NPP against VOD-AGB and PAN-AGB. Figure 2a–c show comparisons between the fully dynamic LPJ-GUESS simulation and the TF-realizations in the downstream AGB with the two empirical datasets, as well as the comparison between the two empirical datasets. The analysis is limited to the area shared between VOD-AGB and PAN-AGB (Supplementary Fig. S4a).

At the global scale, the agreement between the TF-realizations and the VOD-AGB increases slightly in terms of spatial correlation (Fig. 2a) and magnitude (RMSE) (Fig. 2b), compared to the fully dynamic model simulation. The IoA, an evaluation metric that combines the spatial correlation and the magnitude, also shows a small increase at the global scale (Fig. 2c). However, the fully dynamic simulation is already at or very close to the baseline knowledge (the agreement between the two independent empirical datasets), suggesting that the increase in agreement should be interpreted with caution, and may not represent improvements in prediction accuracy.

The agreement over tundra and arctic shrub lands improves markedly when replacing simulated NPP with refined NPP, but the improvement is weaker or slightly negative in other global land cover classes. The tundra and arctic shrubland land cover class also represent the region where the fully dynamic simulation shows the largest room for improvement in comparison to the baseline knowledge (Fig. 2c).

Globally, the agreement between the two empirical datasets (the baseline knowledge) is generally lower for all evaluation metrics (spatial correlation, RMSE and IoA, black segments in Fig. 2a–c) than the agreement between either of the two empirical datasets and the fully dynamic model simulation and the TF-realizations replacing NPP. Similar to the global analysis, where fully dynamic simulation and the TF-realizations showed agreements that exceed the baseline knowledge, most land cover classes show low baseline knowledge in comparison to the model and TF performance, with the tundra and arctic shrubland land cover class as an exception. In addition, the lowest IoA between empirical datasets is found over tropical forest, mainly caused by large RMSE rather than the spatial correlation (Fig. 2). Also notable is the low correlation between the empirical datasets over extra-tropical forest.

Spatially (Fig. 2d,e) the largest improvements of TF-realizations when replacing the simulated NPP with the refined NPP datasets are found in the high latitudes, where the fully dynamic LPJ-GUESS simulation tends to overestimate NPP (Supplementary Fig. S5). Tropical forests show a weaker improvement or reduction in the agreement when replacing NPP, whereas arid regions and the northern boreal forest line show a tendency towards a decreased agreement after replacing NPP.

Taken together, replacing the simulated NPP with NPP derived from empirical datasets leads to relatively small improvements in the predictions of AGB which can be caused by large uncertainties in the AGB datasets, large uncertainties in the GPP or NPP datasets or the vegetation turnover rate of the model.

### Soil carbon

**Figure 3:**
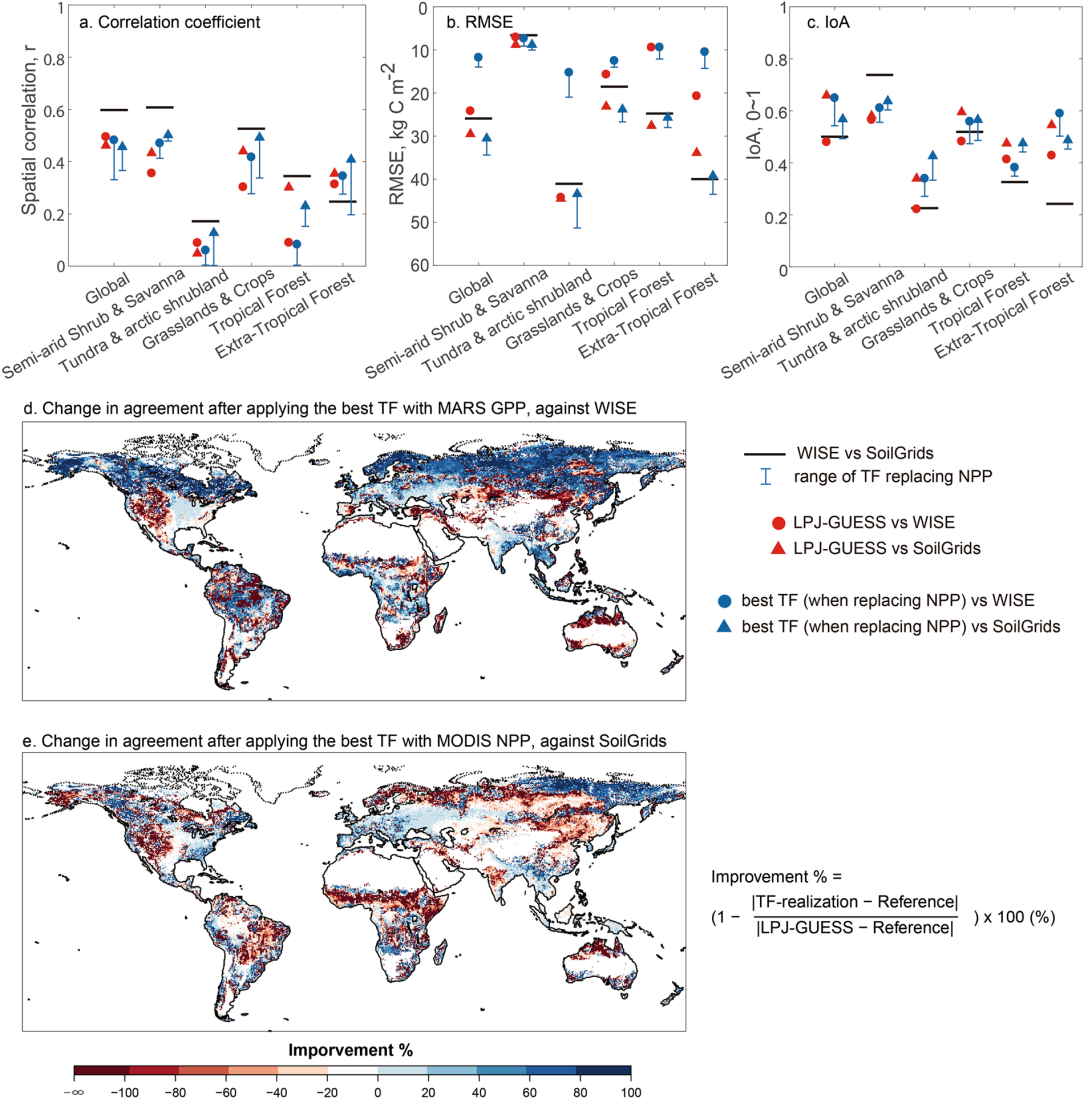
Comparison of spatial soil carbon (representing 2000s) with two independent contemporary empirical datasets (panel a–c) over six land cover classes (Supplementary Fig. S2). Black line segments show the comparison between two empirical datasets of soil carbon. Output from LPJ-GUESS simulation before replacing NPP is marked as red. TF-realizations by replacing simulated NPP with the refined NPP derived from MODIS NPP and FLUXCOM GPP are marked as blue. Error bars show the range of results from replacing simulated NPP with the five refined NPP. Panel d and e show improvement of the best TF-realizations (using FLUXCOM MARS GPP and MODIS NPP) in magnitude for soil carbon comparing to WISE and SoilGrids at 2 m depth, respectively.

Overall, the TF-realizations and the fully dynamic simulation show weak agreement with the two empirical datasets of soil C to two-meter depth (Fig. 3a–c). The improvement of the TF-realization over the fully dynamic simulation in estimating soil C and its spatial pattern is negligible, or the agreement is even reduced. Similarly, over different land cover classes, the improvement is weak, or the agreement is slightly reduced. However, on the other hand, the agreement between the two soil C datasets is also low, limiting the opportunities for any model improvement. The analysis is limited to the shared area between the WISE and SoilGrids datasets (Supplementary Fig. S4b).

Similar to the AGB analysis, the IoA between simulated soil C and either one of the empirical datasets is better than the agreement between the independent empirical datasets over the different land cover classes (except of over semi-arid shrublands and savannas; Fig. 3c). The agreement between empirical datasets and TF-realizations and the fully dynamic simulations as well as between empirical datasets themselves show markedly low agreement over tundra and arctic shrubland, extra-tropical forest and tropical forests (Fig. 3a–c). Over these land cover classes, the best of the TF-realizations (for WISE, using MARS GPP, for SoilGrids, using MODIS NPP) show opposite signs of change in agreement (impair or improve) when comparing to the two independent empirical soil C datasets (Fig. 3d,e). The analysis of soil C highlights negligible improvement of the model-data agreement is due to large uncertainties in the soil carbon datasets, the GPP or NPP datasets, or the soil turnover rate of the model.

Overall, however, our analysis suggests that our ability to evaluate model performance in simulating soil C is limited. While the fully dynamic model and products of the model data fusion, the TF-realizations, show lower agreement to the empirical soil C datasets than they do for vegetation C (AGB), also the agreement between the empirical datasets (the baseline knowledge) is overall weak.

### Net land C flux

**Figure 4:**
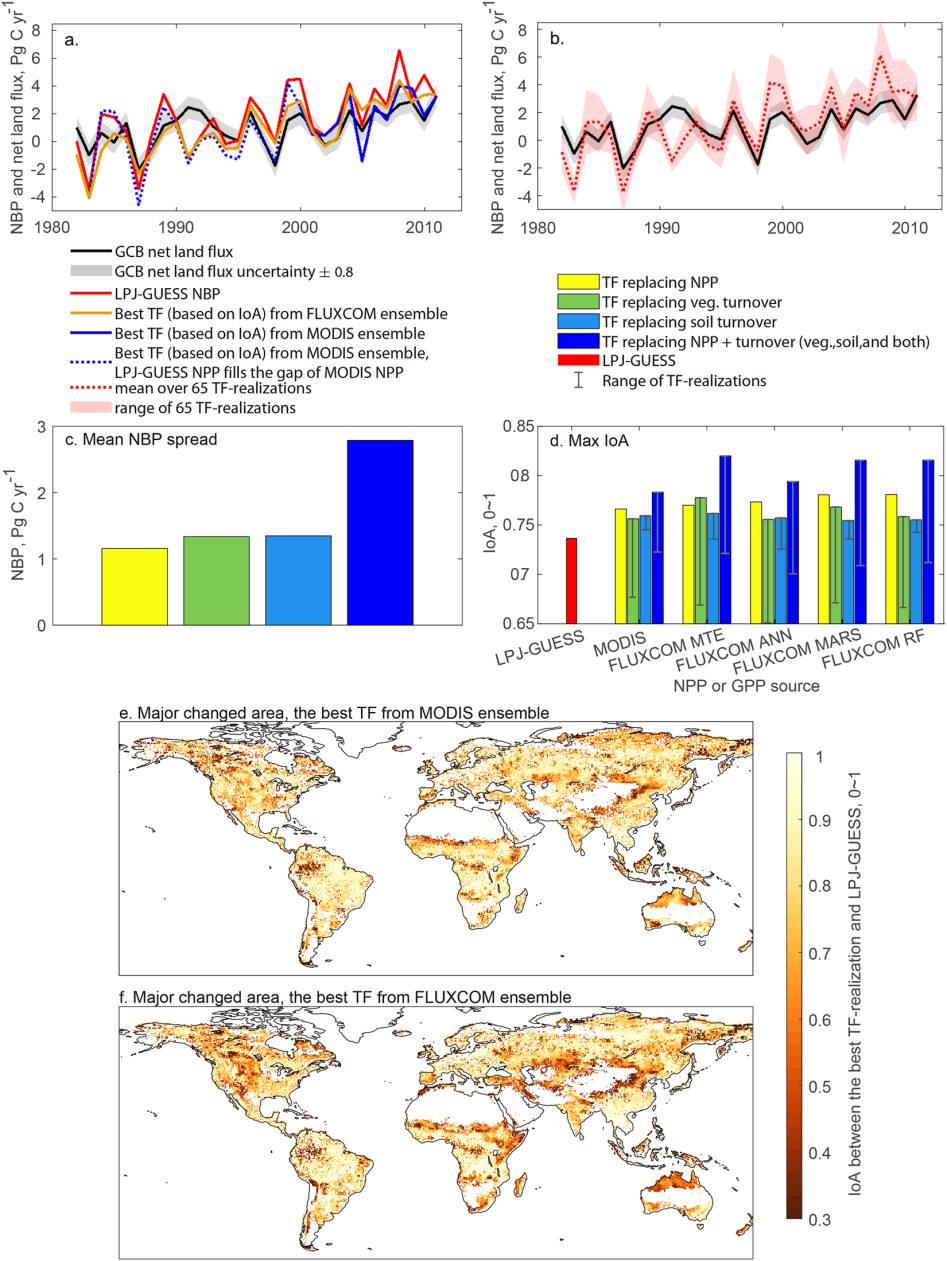
Global annual net land flux and NBP during 1982–2011. (**a**) lines show the temporal pattern of the net land flux derived from LPJ-GUESS (red), the best TF-realization (based on IoA) when using MODIS NPP to correct NPP and C turnover (blue), and the best TF-realization (based on IoA) when using FLUXCOM GPP to correct NPP and C turnover (orange). (**b**). The red shaded area shows the range of 65 TF-realizations (5 for replacing NPP only, 10 for replacing vegetation turnover, 10 for replacing soil turnover, and 40 for replacing NPP and turnover, including vegetation, soil and both turnovers). The net land flux from GCB in (**a**,**b**) is shown in black lines with ± 0.8 Pg C uncertainty range (grey shaded area). (**c**) shows the NBP spread that arises from correction of NPP, vegetation turnover, soil turnover, and the combination of them. (**d**) Bars show maximum IoA when comparing TF-realizations to GCB data in four categories: replacing NPP only (yellow), replacing vegetation turnover only (green), replacing soil turnover only (light blue) and replacing NPP and turnover (dark blue). The error bars show the range for each category TF-realizations. The red bar shows the fully dynamic simulation. All the three statistical evaluation metrics can be found in Supplementary Fig. S6. (**e**,**f**) the dark red area (low IoA) shows the major area of change when applying the best TF-realization using MODIS NPP and FLUXCOM GPP, respectively, in comparison with the fully dynamic LPJ-GUESS simulation.

Apart from analyzing the impact of upstream influx of C on vegetation and soil C stocks, we also explored the effect of C-influx, vegetation and soil C turnover rate on net land-atmosphere C exchange. We used the net biome production (NBP), the net exchange of C between ecosystems and the atmosphere, and compared to the Global Carbon Budget (GCB) net land C flux^36^ over the period that is the common period for FLUXCOM products, 1982–2011 (Fig. 4a). NBP fluxes presented integrate the shared area between VOD-AGB, FLUXCOM GPP, MODIS NPP, SoilGrids and WISE soil C (Supplementary Fig. S3c). PAN-AGB has lower spatial coverage than the other datasets and dynamically simulated vegetation turnover rates are used instead of empirically derived turnover rates in grid cells that are only missing in the PAN-AGB dataset. Only one refined NPP dataset is used for each TF-realization; i.e., the same NPP data is used for correcting C-influx and vegetation and soil C turnover rates. Turnover rates, in turn, are represented by two datasets each, resulting in four TF-realizations in total for each NPP dataset.

Replacing NPP, vegetation and soil C turnover within the TF-realization leads to a minimal effect on predicted NBP, with a total ensemble spread of about 2.8 Pg C per year (Fig. 4b). The ensemble NBP spread arises mainly from the joint effect of variations in NPP, vegetation turnover rate, and soil turnover rate (Fig. 4c). Some of the ensemble members show a higher agreement with the GCB net land flux than the fully dynamic model does (Fig. 4a). The IoA between the fully dynamic simulated NBP and the GCB net land flux is 0.737. The best TF-realization (according to the highest IoA = 0.820, an improvement of 0.083) is based on the FLUXCOM MTE GPP dataset, which was used to derive NPP, vegetation turnover rate when combined with VOD-AGB, and soil turnover rate when combined with WISE (Fig. 4d). For this TF-realization, replacing vegetation turnover rate leads to the largest improvement in IoA (0.817, improvement: 0.080), followed by NPP (0.778, improvement: 0.041), and soil C turnover rate (0.762, improvement: 0.025). TF-realizations based on the other FLUXCOM GPP products and MODIS NPP all show the largest improvement from replacing C-influx alone, and half of them show the second largest improvement from vegetation C turnover rate and soil C turnover rate.

Spatially, NBP changes of the best TF-realization, when compared to the fully dynamic simulation, are quite evenly distributed across biomes with a tendency towards larger changes in semi-arid and tropical ecosystems (the dark red areas in Fig. 4e,f).

## Discussion

Empirical global datasets of terrestrial ecosystem productivity are currently widely used to benchmark models, and as a basis for inferring trends and patterns in the response of the biospheric carbon cycle to global change. Our results using an emulator of a comprehensive and widely-used biosphere model suggest that we are approaching a point where only limited further understanding of land carbon cycle simulations is gained by constraining models based on the present-generation of such global datasets. After replacing simulated NPP with empirical datasets of GPP and NPP, we found small improvements in predicted AGB and soil C storage in comparison with independent contemporary empirical datasets at the global scale. Regionally, TF-realization AGB and soil C show an increased agreement with empirical data at high latitudes. Limitations to the improvement that is possible when replacing C-influx in the dynamic model with observation-based datasets can be imposed by (i) biases in the empirical GPP and NPP datasets, (ii) biases in the data on AGB and soil C storage that was used as reference, (iii) biases in vegetation and soil C turnover in the dynamic model, or (iv) a combination of all.

The biases in empirical GPP and NPP datasets are difficult to assess. The FLUXCOM products originate from a relatively sparse network of flux towers^39^ with GPP being estimated based on measured fluxes of the net C exchange^40^. The spectral reflectance measurements underlying the MODIS NPP dataset are affected by cloud cover^41^ and other factors. Both products rely on modelling for upscaling and interpolation forced by weather data that originate from a sparse network of weather stations^42^. The evaluation data (i.e., PAN-AGB and VOD-AGB, WISE and SoilGrids soil C, and GCB net land C flux) derived from field measurements or inventories, subsequently are integrated by using numerical model, machine learning or other statistical methods, which inevitably contain uncertainties in their method design and processing. The approach adopted here, where model-data agreement is presented in relation to data-data agreement represent an attempt to highlight these uncertainties.

The analysis is based on a single model, a second generation individual based DGVM. While our approach allows us to modify C-influx and the turnover of vegetation and soil C, the structure and the allocation to sub-pools and the relative transfer rates between sub-pools within the vegetation or the soil are preserved in the traceability framework. Other models simulate the allocation and transfer between sub-pools differently and it could be worthwhile to perform future studies using a single or several alternative models to better understand differences between models. However, a multi-model study would likely not change the main results and conclusions of this study.

Our analysis also relates the scores of predicted C storage against the score of the same metric but between two independent empirical datasets. Overall, for both AGB and soil C storage, the independent contemporary empirical datasets show lower agreement than the model-data predictions for any of the datasets suggesting a rather high uncertainty in one of the independent empirical datasets (or both). The soil C datasets, in particular, show very high disagreements, which is consistent with previous model-data comparisons of soil C stock^43^. Hence, the potential to infer a model’s predictive skill or a model’s improvement against the contemporary vegetation and soil C datasets is currently small.

The largest improvement in the agreement between the predicted NBP and the GCB net land C flux was achieved by correcting vegetation and soil C turnover together with C-influx. The generally low agreement between contemporary empirical datasets identified here limits our confidence in inferring what processes in the simulated terrestrial C cycle causes the largest share of overall uncertainty. Our results do, however, indicate that replacing both C-influx and turnover rates with products derived from empirical datasets may lead to large changes in global NBP, vegetation and soil C stocks in the LPJ-GUESS model. Simulated turnover rates of vegetation and soil C are highly dependent on environmental forcing and experimental design. Vegetation turnover arises from multiple factors: disturbances modify the general vegetation C turnover rates that are determined by ecosystem structure, which in turn is influenced by the environment, history, and management. Soil radiocarbon syntheses have revealed that soil C is on average quite old and ESMs underestimated the mean age of soil C by six fold^44^. While the dynamic ecosystem model includes dynamic representations of recalcitrant or “passive” soil C stocks with centennial-scale residence times, this “old” carbon is stored during initialization of the model and then remains relatively inert. The initialization process we adopted here approximates the carbon that would be stored in an environment that resembles the climate between years 1901–1930 with atmospheric CO_2_ concentrations of the year 1901, and it is unclear to what extent such environmental conditions allows for accurate initialization of “old” carbon. Another uncertainty in the simulations is caused by the land cover and land use change, which is represented by a single dataset^20^. Recent studies suggest that time variant large-scale land use datasets may not include important smaller-scale disturbances^45,46^ and the degree to which past land use (before 1901) influences vegetation and soil carbon storage is unclear^47,48^. Hence it remains unclear to what extent forcing data and experimental design contribute to model-data disagreement of both soil and vegetation C storage. Considering the considerable disagreement in empirical datasets for evaluation, it also remains questionable to what extent comparisons between simulated and empirical datasets on land C storage informs us on a model’s ability to simulate changes in land C storage in the modern historical era and in the future.

## Conclusion

By emulating the carbon cycle in terms of responsible processes, stocks and fluxes of a comprehensive and widely-used global biosphere model, we combined the strength of the model in representing processes, with the richness of observational data informing underlying empirical global datasets on ecosystem carbon storage and influx. The resulting tool allowed us to identify those processes that are poorly constrained by the empirical data, providing guidance for model improvement, but also highlighting limitations in the potential for current datasets to constrain models.

Overall we found only small improvements in C storage estimates by correcting the influx of C through GPP or NPP. Also correcting turnover rates led to a large spread in estimated NBP, where some combinations in the empirical datasets used for the corrections led to a better agreement with the GCB net land flux. However, the overall low agreement between independent contemporary empirical datasets on vegetation and soil C storage leaves little room for detectable and robust model improvements. We conclude that differences between state-of-the-art empirical datasets, and our current inability to evaluate them, limits precise inference of uncertainties in simulations of the land C cycle through model-data comparisons.

## Supplementary information

Supplementary information

## Data Availability

Upon publishing of the manuscript all data displayed in result section will be made publically available on the DataGURU server (https://dataguru.lu.se/).
