# Supplementary material for: Approaching the potential of model-data comparisons of global land carbon storage
Source: Sci Rep. 2019 Mar 4;9:3367. doi: 10.1038/s41598-019-38976-y (PMC6399261; doi:10.1038/s41598-019-38976-y)
Supplement: Supplementary file 1 — Supplementary information [file 41598_2019_38976_MOESM1_ESM.docx]

# Supplementary Materials

**Approaching the potential of model-data comparisons of global land carbon storage**

**Zhendong Wu^1,2,*^, Gustaf Hugelius^3,4^, Yiqi Luo^5^, Benjamin Smith^1,6^, Jianyang Xia^7, 8^, Rasmus Fensholt^2^ , Veiko Lehsten^1,9^ and Anders Ahlström^1,3^**

**Table S1.** Independent contemporary data sets of land flux, AGB and Soil C stock. Note: The time period 2000s indicates the data cannot be attributed to a specific year, because the data was derived from input data from different years in 2000s.

| Dataset referred as | Type | Spatial resolution | Time period | Reference |
| --- | --- | --- | --- | --- |
| GCB | Land flux | One Global value | 1959-2015 | Le Quéré et al. (2016) |
| PAN-AGB | AGB | 0.01 degree | 2000s | Avitabile et al. (2016) |
| VOD-AGB | AGB | 0.25 degree | 1993-2012 | Liu et al. (2015) |
| WISE2m | Soil C stock | 0.083 degree | 2000s | Batjes, (2016) |
| SoilGrids2m | Soil C stock | 0.05 degree | 2000s | Hengl et al. (2014) |

**Figure S1.** Comparison of the simulated (red) and TF-realization (green) global carbon stock sizes for each stock. The R^2^ coefficient of determination between the simulated and TF-realization total carbon stock sizes reach 0.998.


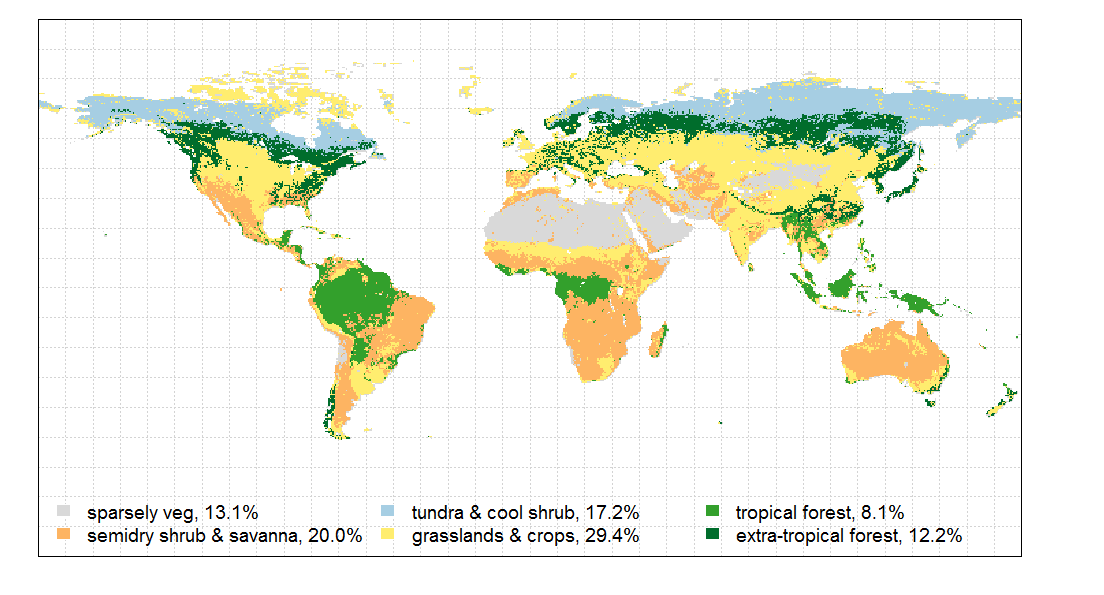


**Figure S2.** Map of land cover classes. The source of the data derived from Ahlström et al. (2015). The percentage values show the fraction of each land cover class of terrestrial area (excluding Greenland).


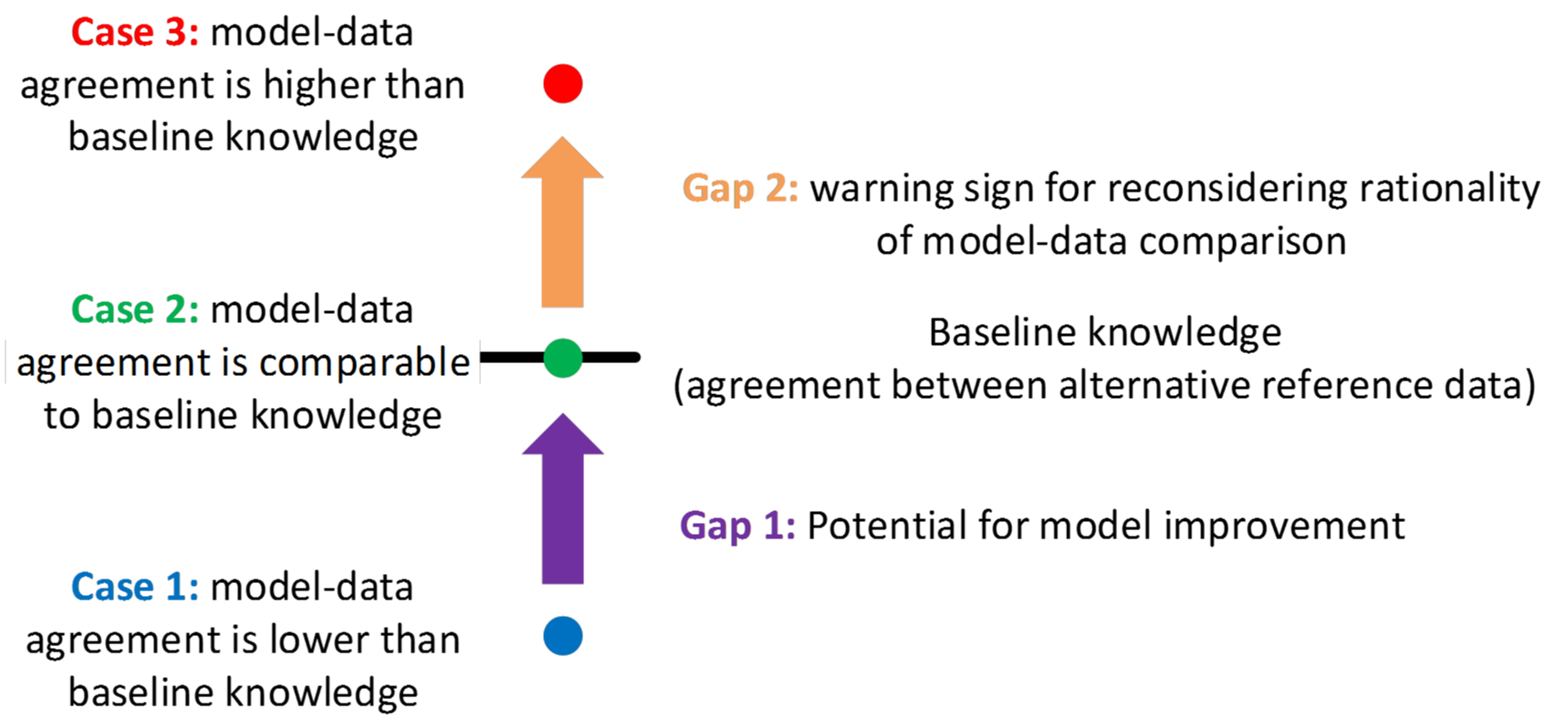


**Figure S3.** Conceptual schematic of possible situations in model-data comparison. Black horizontal line shows the agreement between alternative reference data, which here represents our current baseline knowledge. Three situations are possible: Case 1 (blue dot), model-data agreement is lower than the baseline knowledge, the gap between them (Gap1, purple arrow) indicates the clear potential for model improvement. Case 2 (green dot), the agreement is around the baseline knowledge. Case 3 (red dot), the model-data agreement is higher than the baseline knowledge, the gap between them (Gap 2, orange arrow) shows warning sign for reconsidering rationality of the model-data comparison.

**Figure S4.** The common area for both TF-realizations and reference data masked as grey, and used for analysis of AGB (a), soil C (b) and NBP (c).

**Figure S5.** spatial comparison of annual mean NPP between empirical estimates and LPJ-GUESS simulation over the period (2000-2011). Map a shows the bias between MODIS NPP and LPJ-GEUSS. Maps b-e show the bias between translated FLUXCOM NPP (by removing the plant respiration from MET, ANN, MARS and RF GPP) and LPJ-GUESS. Panel f shows full matrixes of comparisons of Pearson correlation coefficient between empirical NPP and LPJ-GUESS NPP.

**Figure S6.** The comparison between TF-realization NBP and GCB net land flux during 1982-2011. (a-c) show temporal correlation, RMSE and IoA when comparing the fully dynamic simulation and TF-realization to GCB data. The red segment which indicates the fully dynamic simulation, and the dotted segments represent TF-realization when replacing C flux only. The boxplots show the result of TF-realizations when replacing C-influx, vegetation and soil C turnover. On each box, the central mark indicates the median, and the bottom and top edges of the box indicate the 25th and 75th percentiles, respectively. The bottom and top whiskers show the minimum and maximum value. The comparison is further split into four categories: replacing NPP only (yellow), replacing vegetation turnover only (green), replacing soil turnover only (light blue) and replacing NPP and turnover (dark blue). The error bars show the range for each category TF-realizations. The red bar shows the fully dynamic simulation. The comparison is shown as in the temporal correlation (d), RMSE (e) and IoA (f).

### **1. Theoretical framework**

The traceability framework is a model emulator, which represents the carbon cycle in the dynamic ecosystem model (e.g. LPJ-GUESS) and preserves the model structure and functioning in space and time. Carbon storage dynamics can be mathematically represented by a matrix model (Jiang et al. 2018; Luo et al., 2015; Luo et al., 2003; Xia et al., 2013) as:

$X^{'}\left( t \right)=B(t)U\left( t \right)-A(t)\xi\left( t \right)KX\left( t \right)$ (1)

where $B(t)$ is a vector of the partitioning coefficients of the photosynthesized carbon into different plant stocks, $U\left( t \right)$ is the carbon fixed via plant photosynthesis (NPP), $A(t)$ is a matrix of transfer coefficients of carbon exit from one stock into another stock, $\xi\left( t \right)$ is a diagonal matrix of environmental scalars, $K$ is a diagonal matrix of the first-order baseline turnover or decomposition rate. $X\left( t \right)$ is a vector of carbon stock sizes.

The traceability framework can be used to decompose the terrestrial C cycle of an ecosystem model into a few traceable components (e.g. NPP, allocation coefficients, transfer coefficients etc.). And traceability framework has been used to identify uncertainty in modeling terrestrial carbon cycle, e.g. Ahlström et al., (2015) applied the traceability framework to LPJ-GUESS to quantify the relative roles of ecosystem C cycle processes (i.e. NPP, vegetation turnover, and soil decomposition) in contributing to future C uptake uncertainties under different climate change scenarios. Besides, traceability framework has been able to implement pool-based data assimilation and improve model performance. For instance, the capacity of ecosystem models such as the TECO model (Shi et al., 2015, Du et al., 2017) and the CLM-CASA model (Hararuk et al., 2014) in projecting C pool and flux dynamics have been substantially improved via data assimilation using matrix representation of these models.

## Text 2. Model and simulation protocol

We used the dynamic ecosystem model LPJ-GUESS (Smith et al., 2001, Smith et al., 2014) to estimate carbon fluxes and carbon storage dynamics over a gridded representation of the land surface. The simulation was initialized with a 1020 years spin-up, to equilibrate soil and vegetation stocks. During the initialization de-trended 1901-1930 climate forcing fields were applied together with constant CO_2_ concentration and nitrogen deposition representing year 1901. After the initialization, time-varying historical CO_2_ (Keeling et al., 2009), nitrogen deposition (Lamarque et al., 2011) and climate data from the CRUNCEP version 7 (Wei et al., 2013) were applied. The simulation used 20 patches per grid cell, starts 1901 and ends 2014. Land use data came from Hurtt et al. (2011).

## Text 3. TF implementation

### 3.1 NPP harmonization

Harmonized time series of NPP covering the period 1901 through 2011 was created by merging simulated GPP and NPP with FLUXCOM GPP and MODIS NPP products. The corrections and adjustments in the harmonization procedure were chosen and designed to avoid abrupt shifts in mean NPP, its interannual variations and in the trend.

The method preserves the simulated NPP trend but uses the mean NPP and the interannual variability (deviations from long term trend) of the empirical datasets. FLUXCOM GPP products were translated to NPP by removing the simulated plant respiration fraction of GPP.

The trends of the empirical NPP datasets were replaced with the simulated trend:

$detrend\left( {NPP}^{obs} \right)={NPP}^{obs}-trend\left( {NPP}^{obs} \right)$, (2)

${NPP}^{obs \_trend}=detrend\left( {NPP}^{obs} \right)+trend({NPP}^{LPJG})$, (3)

Where ${NPP}^{obs}$ is the empirical NPP (MODIS NPP or FLUXCOM NPP after removing plant respiration), and ${NPP}^{LPJG}$ is the simulated NPP.

To avoid abrupt shifts in NPP the simulated NPP time series was bias corrected to represent the mean NPP of the empirical NPP products over their overlapping time period (com period: 1982-2011 for FLUXCOM, and 2000-2014 for MODIS)

${NPP}_{t}^{LPJG\_bc}= {NPP}_{t}^{LPJG}*( \bar{{NPP}_{com period}^{obs\_ trend}}/\bar{{NPP}_{com period}^{LPJG})}$, (4)

where ${NPP}_{t}^{LPJG\_bc}$ is the bias corrected NPP for year *t* and ${NPP}_{t}^{LPJG}$ is the simulated the NPP from 1901 to 2014.

The interannual variability of the simulated NPP was also corrected to match the variability of the empirically derived NPP products. This was done by correcting the standard deviations of the annual anomalies from the long term mean.

${NPP}^{LPJG\_bc\_IAV}=detrend\left( {NPP}^{LPJG\_bc} \right)/ sd(detrend\left( {NPP}_{com period}^{LPJG\_bc} \right))*sd(detrend\left( {NPP}_{com period}^{obs} \right))+trend\left( {NPP}^{LPJG\_bc} \right)$*,* (5)

Where *sd* denotes the standard deviation.

The harmonized NPP datasets were created by merging the simulated NPP time series after corrections of mean NPP and interannual variability (${NPP}^{LPJG\_bc\_IAV}$) and the trend corrected empirically derived NPP (${NPP}^{obs \_trend}$).

### 3.2. Turnover rate correction

Simulated turnover rates of vegetation and soil C are adjusted based on the apparent mean turnover rate *aMTR*:

$aMTR= \bar{NPP}/\bar{Cpool}$ (6)

where $Cpool$ is vegetation or soil carbon storage. For vegetation C turnover rate $aMTR$ was calculated over the period 1993-2012, in both simulated and empirical data. For soil C, where the empirical datasets are not resolved in time, the same period 1993-2012 was used to calculate average $aMTR$ on the simulated data. The apparent turnover rate at year t (*aTR_t_*) of the TF realizations was bias-corrected using relative anomalies:

${aTR}_{t}^{bc}={aTR}_{t}*{aMTR}^{obs}/{aMTR}^{LPJG}$ (7)

The vegetation C turnover rates are based on above ground biomass (excluding roots in the simulated data), but the correction was also applied on the root C turnover rate. The soil C turnover rate was based on all soil C to two meters depth in the empirical datasets, and applied uniformly to all soil C stocks in the TF realizations.

The turnover rates described here are apparent turnover rates, calculated using available C-influx dataset. A potential trend in C-influx would bias the calculations of turnover rates described here, since C-influx ~= C-efflux from soil and vegetation C stocks. However, the calculations are performed in the same way on the simulated data and the empirical data, which implies that this bias is at least partly offset under the assumption that the dynamic model simulates a reasonably realistic trend in C-influx.

### 3.3. TF spin-up

Initial state condition of TF-realization was calculated by two fundamental components of traceability framework: (i) net primary productivity (NPP), i.e. $U(t)$, and (ii) ecosystem residence time ($\tau_{e}$), i.e. ${(A(t)\xi\left( t \right)K)}^{-1}*B(t)$. $\tau_{e}$ is codetermined by transfer coefficients ($A$), environmental scalar ($\xi$), baseline turnover rate ($K$), and allocation coefficients ($B$). By letting $X^{'}\left( t \right)$ in equation 1 equal zero, the steady state ecosystem C storage ($X_{ss}$, the maximum C amount that an ecosystem can potentially store) can be estimated as:

$X_{ss}\left( t \right)= {(A(t)\xi\left( t \right)K)}^{-1}*B(t)U\left( t \right)$ (8)

We used traceable components from the last 90 years of spin-up to solve the $X_{ss}\left( t \right)$ and used as in initial state condition for TF-realization.

# Supplement Reference

AHLSTRÖM, A., XIA, J., ARNETH, A., LUO, Y. & SMITH, B. 2015. Importance of vegetation dynamics for future terrestrial carbon cycling. *Environmental Research Letters,* 10**,** 054019.

AVITABILE, V., HEROLD, M., HEUVELINK, G., LEWIS, S. L., PHILLIPS, O. L., ASNER, G. P., ARMSTON, J., ASHTON, P. S., BANIN, L. & BAYOL, N. 2016. An integrated pan‐tropical biomass map using multiple reference datasets. *Global change biology,* 22**,** 1406-1420.

BATJES, N. H. 2016. Harmonized soil property values for broad-scale modelling (WISE30sec) with estimates of global soil carbon stocks. *Geoderma,* 269**,** 61-68.

DU, Z. G., ZHOU, X. H., SHAO, J. J., YU, G. R., WANG, H. M., ZHAI, D. P., XIA, J. Y. & LUO, Y. Q. 2017. Quantifying uncertainties from additional nitrogen data and processes in a terrestrial ecosystem model with Bayesian probabilistic inversion. *Journal of Advances in Modeling Earth Systems,* 9**,** 548-565.

HARARUK, O., XIA, J. Y. & LUO, Y. Q. 2014. Evaluation and improvement of a global land model against soil carbon data using a Bayesian Markov chain Monte Carlo method. *Journal of Geophysical Research-Biogeosciences,* 119**,** 403-417.

HENGL, T., DE JESUS, J. M., HEUVELINK, G. B. M., GONZALEZ, M. R., KILIBARDA, M., BLAGOTIC, A., SHANGGUAN, W., WRIGHT, M. N., GENG, X. Y., BAUER-MARSCHALLINGER, B., GUEVARA, M. A., VARGAS, R., MACMILLAN, R. A., BATJES, N. H., LEENAARS, J. G. B., RIBEIRO, E., WHEELER, I., MANTEL, S. & KEMPEN, B. 2017. SoilGrids250m: Global gridded soil information based on machine learning. *Plos One,* 12.

HURTT, G., CHINI, L. P., FROLKING, S., BETTS, R., FEDDEMA, J., FISCHER, G., FISK, J., HIBBARD, K., HOUGHTON, R. & JANETOS, A. 2011. Harmonization of land-use scenarios for the period 1500–2100: 600 years of global gridded annual land-use transitions, wood harvest, and resulting secondary lands. *Climatic Change,* 109**,** 117-161.

KEELING, R., PIPER, S., BOLLENBACHER, A. & WALKER, J. 2009. Atmospheric carbon dioxide record from Mauna Loa. ESS-DIVE (Environmental System Science Data Infrastructure for a Virtual Ecosystem); Oak Ridge National Laboratory (ORNL), Oak Ridge, TN (United States).

LAMARQUE, J.-F., KYLE, G. P., MEINSHAUSEN, M., RIAHI, K., SMITH, S. J., VAN VUUREN, D. P., CONLEY, A. J. & VITT, F. 2011. Global and regional evolution of short-lived radiatively-active gases and aerosols in the Representative Concentration Pathways. *Climatic change,* 109**,** 191-212.

LE QUÉRÉ, C., ANDREW, R. M., CANADELL, J. G., SITCH, S., KORSBAKKEN, J. I., PETERS, G. P., MANNING, A. C., BODEN, T. A., TANS, P. P. & HOUGHTON, R. A. 2016. Global carbon budget 2016. *Earth System Science Data,* 8**,** 605.

LIU, Y. Y., VAN DIJK, A. I., DE JEU, R. A., CANADELL, J. G., MCCABE, M. F., EVANS, J. P. & WANG, G. 2015. Recent reversal in loss of global terrestrial biomass. *Nature Climate Change,* 5**,** 470-474.

LUO, Y., SHI, Z., LU, X., XIA, J., LIANG, J., JIANG, J., WANG, Y., SMITH, M. J., JIANG, L. & AHLSTRÖM, A. 2017. Transient dynamics of terrestrial carbon storage: mathematical foundation and its applications. *Biogeosciences,* 14**,** 145.

LUO, Y., WHITE, L. W., CANADELL, J. G., DELUCIA, E. H., ELLSWORTH, D. S., FINZI, A., LICHTER, J. & SCHLESINGER, W. H. 2003. Sustainability of terrestrial carbon sequestration: a case study in Duke Forest with inversion approach. *Global biogeochemical cycles,* 17.

SHI, Z., XU, X., HARARUK, O., JIANG, L. F., XIA, J. Y., LIANG, J. Y., LI, D. J. & LUO, Y. Q. 2015. Experimental warming altered rates of carbon processes, allocation, and carbon storage in a tallgrass prairie. *Ecosphere,* 6.

SMITH, B., PRENTICE, I. C. & SYKES, M. T. 2001. Representation of vegetation dynamics in the modelling of terrestrial ecosystems: comparing two contrasting approaches within European climate space. *Global Ecology and Biogeography,* 10**,** 621-637.

SMITH, B., WARLIND, D., ARNETH, A., HICKLER, T., LEADLEY, P., SILTBERG, J. & ZAEHLE, S. 2014. Implications of incorporating N cycling and N limitations on primary production in an individual-based dynamic vegetation model. *Biogeosciences,* 11**,** 2027-2054.

WEI, Y., LIU, S., HUNTZINGER, D., MICHALAK, A., VIOVY, N., POST, W., SCHWALM, C., SCHAEFER, K., JACOBSON, A. & LU, C. 2013. The North American carbon program multi-scale synthesis and terrestrial model intercomparison project–part 2: environmental driver data. *Geoscientific Model Development Discussions,* 6**,** 5375-5422.

XIA, J., LUO, Y., WANG, Y. P. & HARARUK, O. 2013. Traceable components of terrestrial carbon storage capacity in biogeochemical models. *Global change biology,* 19**,** 2104-2116.
